# Absolute monocyte counts could predict disease activity and secondary loss of response of patients with Crohn’s disease treated with anti-TNF-α drug

**DOI:** 10.1371/journal.pone.0301797

**Published:** 2024-04-10

**Authors:** Jia Hu, Yin Huang, Ruonan Jia, Xuehong Wang, Yongjun Wang

**Affiliations:** 1 Department of Gastroenterology, The Second Xiangya Hospital, Central South University, Changsha, Hunan, China; 2 Research Center of Digestive Disease, The Second Xiangya Hospital, Central South University, Changsha, Hunan, China; University of Montenegro-Faculty of Medicine, MONTENEGRO

## Abstract

**Background:**

Assessing Crohn’s disease (CD) activity is critical for monitoring disease progression. In CD, monocytes could release TNF-α. Thus, it is extremely important to study its role in the disease activity and loss of response to anti-TNF-α biologics.

**Methods:**

In this study, we collected CD patients treated with biologics from January 2017 to May 2022. Indicators associated with disease activity were evaluated by Spearman correlation analysis and Mann-Whitney U test. Specifically, logistic analyses were used to explore the predictors of primary nonresponse (PNR) and secondary loss of response (SLOR) within 1 year of anti-TNF-α agents. In addition, a nomogram was developed for therapeutic effect prediction.

**Results:**

283 patients with CD were identified. Disease activity group, defined as CDAI equal to or greater than 150, had significant elevated absolute monocyte counts than disease remission group based on CDAI score (p = 0.019, Z = -2.354). Logistic analyses showed that absolute monocyte counts could be an independent predictor of 1-year SLOR of anti-TNF-α agents in CD patients (p = 0.013). A nomogram established based on gender, absolute monocyte counts, and hemoglobin could predict SLOR within 1 year of anti-TNF-α agents reliably.

**Conclusion:**

The results of this study support the utility of absolute monocyte counts detecting disease activity and anti-TNF-α therapy effect in patients with CD.

## 1. Introduction

Crohn’s disease (CD), one type of inflammatory bowel disease (IBD), is a chronic and recurrent inflammatory bowel disease [1]. A variety of biomarkers are used to evaluate disease activity [2,3]. Blood biomarkers are the most readily available. Erythrocyte sedimentation rate (ESR) and C-reactive protein (CRP) usually elevated in CD patients, while they are non-specific and increase in many inflammatory responses, such as infections and autoimmune diseases. Besides, Fecal calprotectin and endoscopy are more specific and sensitive to disease activity [4,5], but are expensive and difficult to be used in the clinical practice commonly. Therefore, there is an urgent need for a cost-effective, non-invasive and effective biomarker for CD activity in the clinical practice.

Biologics have been widely used in patients with CD. Anti-tumor necrosis factor α (Anti-TNF-α) medications are representative drug in CD [6,7]. However, approximately one-third of CD patients treated with anti-TNF-α drugs have no response initially, known as primary nonresponse (PNR) [8,9]. Among patients with effective primary treatment, half of them lost effect in the maintenance phase, which is called secondary loss of response (SLOR) [10]. Due to the side effects and high cost of biologics [7,11], early identification of predictors for effective biological therapy can timely optimize treatment, consequently reduce economic burden and achieve optimal outcomes.

White blood cell and its subclasses are routinely examined and are regarded as predictive markers of inflammatory diseases. Monocyte is a kind of white blood cell that originates from progenitor cells in the bone marrow. When encountered with inflammation, monocytes migrate into tissues, then differentiate into macrophages and dendritic cell to play the role of pathogen clearance and anti-inflammation [9,12,13]. Recent studies found that monocytosis is associated with long-term disease severity in both adults and pediatric CD patients [14,15]. In addition, it is also closely related to disease activity in ulcerative colitis and IBD patients with diabetes mellitus [16,17]. However, the relationship between absolute monocyte counts or its percentage and disease activity in CD patients remains uncertain. Surprisingly, monocyte is also related to therapeutic effect of biologics. Study showed that monocyte percentage greater than 8.15% at the time of discontinuing biologics was associated with a high relapse rate within 6 months in IBD patients [18]. Another study included 71 CD patients with anti-TNF-α drugs followed up for 16 months, and it showed a significant difference in monocyte counts between recurrence and remission of CD (p< 0.005) [19]. To date, no research has been presented to explore the role of monocyte in losing of response to biologics in patients with CD.

The aim of the study was to examine the association of absolute monocyte counts and monocyte percentage with CD activity. In the meantime, their predictive value in losing of response of anti-TNF-α drug in CD patients was also verified.

## 2. Methods and materials

### 2.1 Patients, study design and definitions

In this study, we searched electronic medical record system on September 1st, 2023 and collected CD patients treated with biologics from the Second Xiangya Hospital of Central South University from January 2017 to May 2022. The inclusion criteria were: (1) Patients diagnosed with CD. The diagnosis of CD was based on the comprehensive analysis of medical history, clinical characteristics, radiological, endoscopic, laboratory and histological examinations. The diagnosis was made independently by two physicians. If two physicians came to the same conclusion, it was accurately determined whether the patient was diagnosed with CD. If the two physicians draw different conclusions, the third physician determined; (2) Patients with complete absolute monocyte counts and monocyte percentage data; (3) Patients treated with regular biologics; (4) Age of patients from 18 to 70 years old. The exclusion criteria were: (1) Standardized biologics treatment was not performed; (2) Patients with pregnancy, serious underlying diseases, infectious diseases, tumors, serious liver diseases, nephropathy, heart diseases, lung disease and autoimmune disease; (3) Patients with incomplete absolute monocyte counts and monocyte percentage data.

### 2.2 Data collection

Demographic data were collected including sex, age at diagnosis and body mass index (BMI). Disease characteristics such as disease behavior, perianal lesions, previous intestinal surgery, disease activity, and types of biological agents were also included. Besides, laboratory tests including absolute monocyte counts, monocyte percentage, Hemoglobin (Hb), absolute lymphocyte count, absolute neutrophil count, platelet count (PLT), hematocrit (HCT), prothrombin time (PT), activated partial thromboplastin time (APTT), D-Dimer, erythrocyte sedimentation rate (ESR), C-reactive protein (CRP), albumin (ALB) and total bilirubin (TBIL) were involved in the study.

### 2.3 Disease activity

Crohn’s disease activity index (CDAI) is commonly used to evaluate the disease activity of CD in the clinical work, and the CDAI calculation method proposed by Best et al. has been widely used in scientific research [20]. CDAI less than150 is defined as disease remission, and CDAI equal to or greater than 150 is defined as active disease. Further, CDAI from 150 to 220 was defined as mild active disease. CDAI from 221 to 450 was defined as moderate active disease. Greater than 450 was defined as severe active disease. CD activity was evaluated by two experienced physicians based on the CDAI.

### 2.4 Outcomes

CD Patients treated with anti-TNF-α agents including adalimumab and infliximab were included in treatment effect analysis. PNR was defined as loss of response within 14 to 16 weeks treated with biologic agents [21]. SLOR at 1 year was defined that biologics have therapeutic effect at 14 to 16 weeks and loss its role within 1 year.

### 2.5 Statistical analysis

Continuous variables with normal distribution were summarized as mean and standard deviation. While for non-normally distributed variables, median and interquartile range were reported. For categorical variables, frequency and proportion were used to describe. Spearman correlation analysis and Mann-Whitney U test were used to investigate the relationship between absolute monocyte counts or monocyte percentage and disease activity. Indicators with P value less than 0.05 obtained from Mann-Whitney U test can be further included in collinearity test. Indicator with Tolerance greater than 0.1 or variance Inflation factor less than 10 are considered no multicollinearity [22]. Indicators with significant differences between the active disease group and the remissive group and no multicollinearity are included in the multivariate logistic regression model. A p-value less than 0.05 was considered as a significant level in correlation analysis and multivariate logistic regression analyses. Hosmer-Lemeshow was used to evaluate the goodness of fit of the model and p>0.05 indicates that the calibration degree of the model is good [23]. ROC curves of single laboratory indicator and combined indicators were plotted, and the area under curve of ROC curves were calculated and compared between them [24].Univariate logistic and multivariate logistic regression models were performed to identify the predictive factors of PNR and SLOR within 1 year. The results were represented as odds ratio (OR) and its 95% confidence interval (95% CI). In order not to miss variables, p-value < 0.2 was considered statistically significant in univariate logistic regression analysis. A p-value less than 0.05 was considered as a significant level in the multivariate logistic regression analysis. Combined with indicators which could dependently predic SLOR within 1 year, a nomogram for predicting SLOR within 1 year of anti-TNF-α agents in CD patients was created using the ‘rms’ R package. Calibration curve and Hosmer-Lemeshow were used to evaluate the capacity of nomogram for predicting SLOR within 1 year of anti-TNF-α agents in CD patients. The statistical software SPSS (v27.0, IBM) and R software (v4.0.5, https://www.r-project.org/) were applied in this study.

### 2.6 Ethical considerations

This study was approved by the Ethics Committee of the Second Xiangya Hospital of Central South University (NO. LYF2023123). Because collected data was anonymous, all patients do not need informed consent.

## 3. Results

### 3.1 Demographic characteristics

In this retrospective study, a total of 283 CD patients met inclusion criteria were enrolled. The demographic characteristics were summarized in Table 1. We found that the subjects were mostly male (72.4%). The median diagnostic age was 26 years old and the median BMI was 19 kg/m^2^. For the lesion site, L3, represented the ileocolecal lesion, was the most common type with 173 patients (61.1%). Information on the presence of stenosis, penetration, perianal lesions, and previous bowel surgery history was also summarized in the Table 1. There were 224 patients with disease activity, among which most patients were mild activity (n = 117, percent = 42.3%). Other 59 patients were in remission (n = 59, percent = 20.8%). For the type of biologics, it was surprised that adalimumab (n = 106, percent = 37.5%) and infliximab (n = 107, percent = 37.8%) were the most used in clinical practice, both of which were anti-TNF-α agents.

**Table 1:** Demographic characteristics.

	Overall (283)
Gender (%)	
Male	205 (72.4)
Female	78 (27.6)
Age of diagnosis (median ± IQR, years)	26 [20, 34]
Body mass index (median ± IQR, kg/m^2^)	19 [17, 21]
Location (%)	
L1	63 (22.3)
L2	15 (5.3)
L3	173 (61.1)
L1+L4	6 (2.1)
L3+L4	26 (9.2)
Stricture (%)	
Yes	144 (50.9)
No	139 (49.1)
Penetrating (%)	
Yes	70 (24.7)
No	213 (75.3)
Perianal disease (%)	
Yes	106 (37.5)
No	177 (62.5)
Intestinal surgery (%)	
Yes	69 (24.4)
No	214 (75.6)
Activity (%)	
Remission	59 (20.8)
Mild activity	117 (42.3)
Moderate activity	98 (34.6)
Severe activity	9 (3.2)
Type of Biologics (%)	
Adalimumab	106 (37.5)
Infliximab	107 (37.8)
Vedolizumab	23 (8.1)
Ustekinumab	28 (9.9)
Not available	19 (6.7)

### 3.2 Correlation analysis of monocyte and its percentage with disease activity

**Fig 1:**
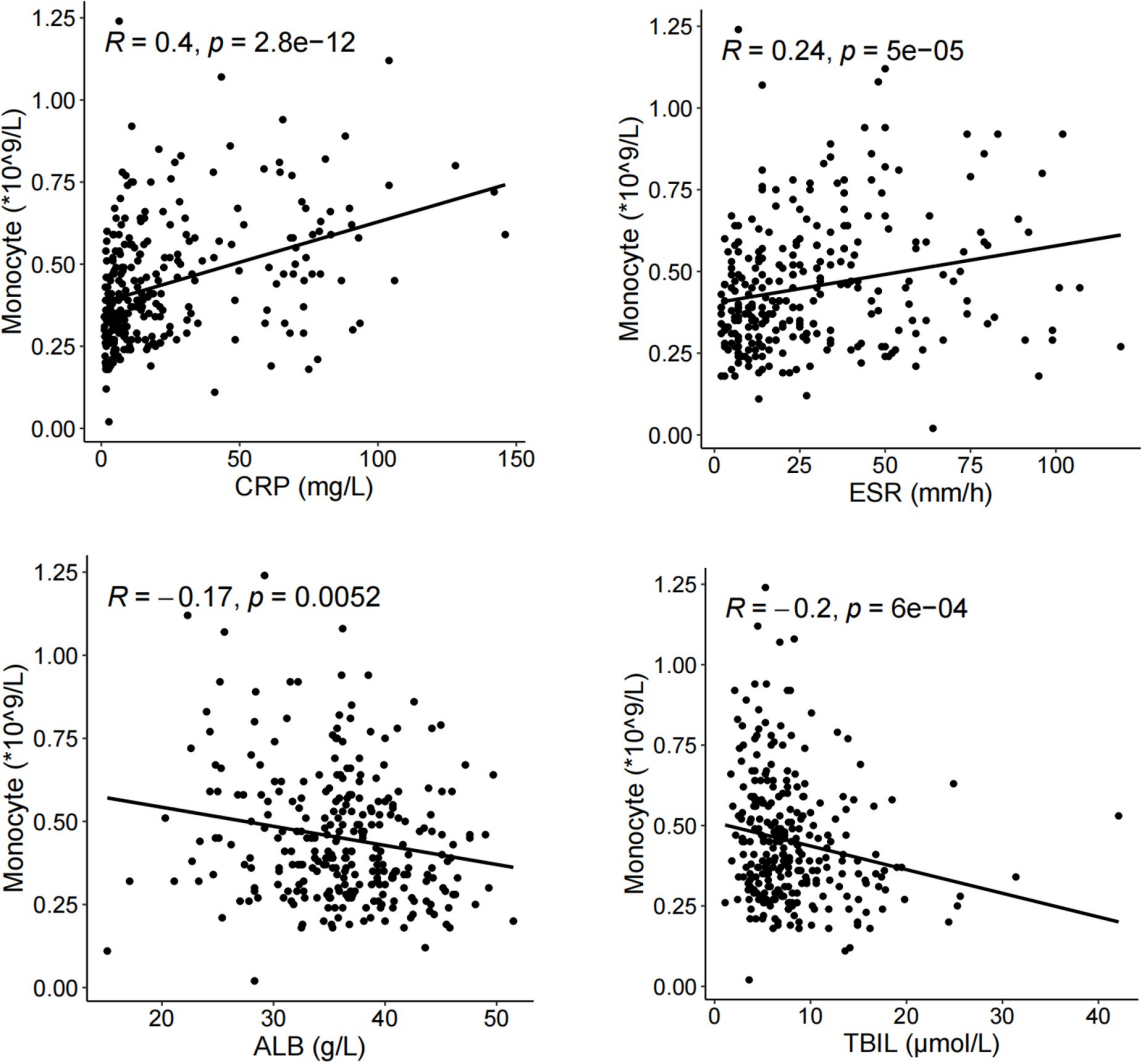
The correlation curve about absolute monocyte counts and biochemical indexes. (a) CRP, (b) ESR, (c) ALB and (d) TBIL. Abbreviation: CRP, C-reactive protein; ESR, erythrocyte sedimentation rate; ALB, albumin; TBIL, total bilirubin.

A number of studies have shown that CRP, ESR, ALB and TBIL are closely related to disease activity in patients with CD. CRP and ESR are positively correlated with disease activity, while ALB and TBIL are negatively correlated with disease activity [15,17,25,26]. Spearman correlation analysis was used to investigate the relationship between absolute monocyte counts and its percentage and disease activity. Fig 1 showed that absolute monocyte counts was significant positive associated with CRP (r = 0.40, p < 0.001) and ESR (r = 0.24, p < 0.001). A negative correlation was observed between absolute monocyte counts and ALB (r = -0.17, p = 0.0052), and TBIL (r = -0.2, p < 0.001). These data suggest that absolute monocyte counts were closely related to CD disease activity.

The relationship between Monocyte percentage and indicators related to disease activity also was evaluated. We detected a positive correlation between monocyte percentage and CRP (r = 0.174, p = 0.003). A negative correlation was found between monocyte percentage and ALB (r = -0.161, p = 0.007), while no evident correlation was observed between monocyte percentage with ESR (r = 0.038, p = 0.529), TBIL (r = -0.057, p = 0.339) (S1 File).

### 3.3 Relationship between laboratory indicators and clinical disease activity

**Fig 2:**
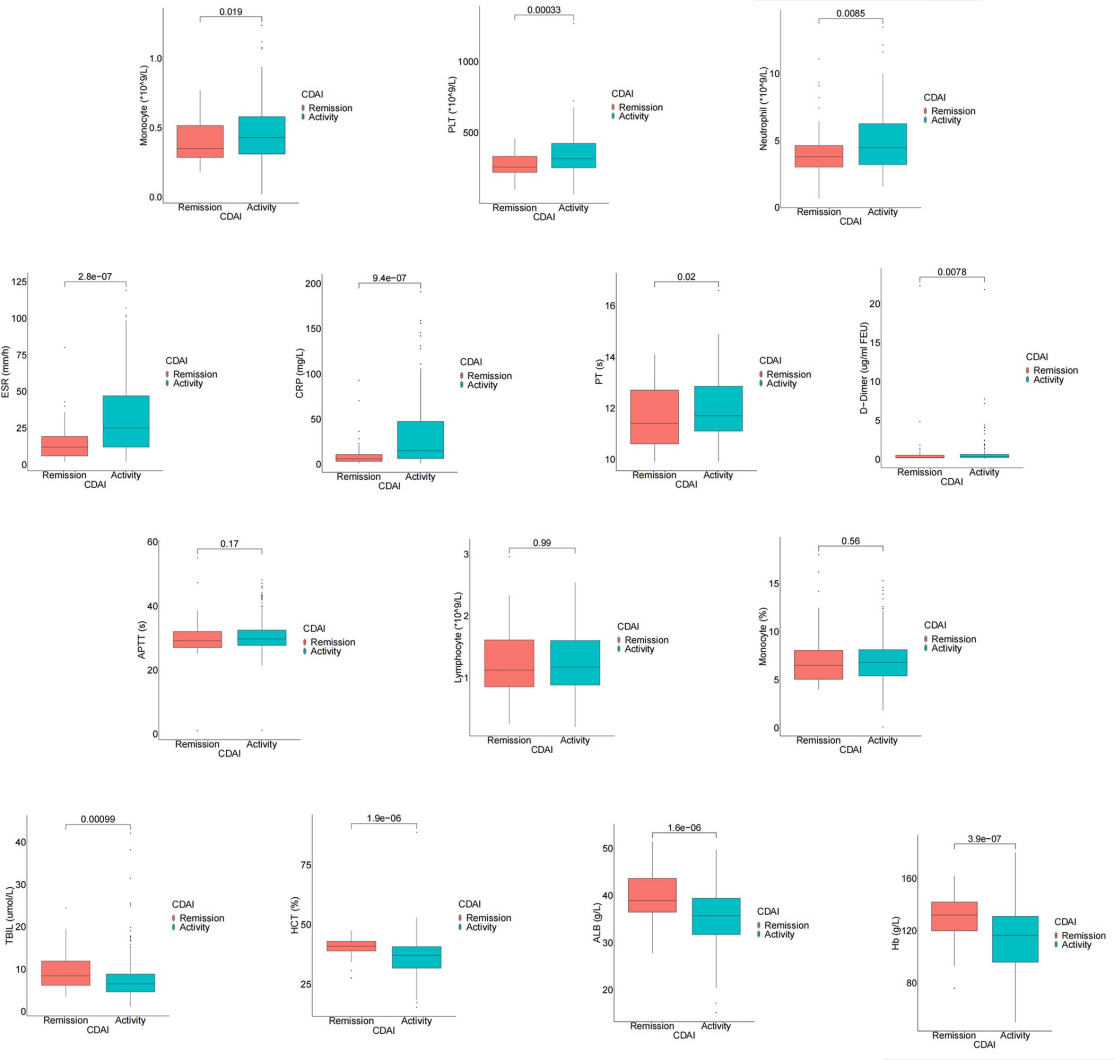
The box plot of laboratory indicators and different CDAI group. Abbreviation: CDAI, Crohn’s Disease Activity Index; Hb, Hemoglobin; PLT, platelet count; HCT: Hematocrit; PT, prothrombin time; APTT, activated partial thromboplastin time; ESR, erythrocyte sedimentation rate; CRP, C-reactive protein, ALB, albumin; TBIL, total bilirubin.

According to CDAI, the subjects were divided into the active disease group and the remissive disease group, and the differences in laboratory indexes between these two groups were further compared. Results showed that compared with disease remission group, absolute monocyte counts, PLT, absolute neutrophil count, ESR, CRP, PT and D-Dimer were significantly increased in the active disease group (p = 0.019, <0.001, 0.0085, <0.001, <0.001, 0.02, 0.0078, respectively). APTT, monocyte percentage and absolute lymphocyte count in the active disease group were not different from those in the remission group (p = 0.17, 0.99, 0.56, respectively). On the contrary, TBIL, ALB, HCT and Hb in active disease group were significantly higher than those in the remission group (p<0.001). In conclusion, Mann-Whitney U test showed that absolute monocyte count, PLT, absolute neutrophil count, ESR, CRP, PT, D-Dimer, TBIL, ALB, HCT, Hb were correlated with disease activity of Crohn’s disease (Fig 2). Further analysis was conducted to determine whether there were differences in absolute monocyte counts and monocyte percentage among the remission group, the mild activity group, the moderate activity group and the severe activity group. S2 File showed that absolute monocyte counts can distinguish between the mild activity group and the remission group (p< 0.01), the moderate activity group and the mild activity group (p<0.05), the severe activity group and the remission group (p<0.01), the severe activity group and the mild activity group (p<0.05). while, there was no significant difference in absolute monocyte counts between the mild activity group and the remission group, the heavy activity group and the moderate activity group. What’s more, there was no significant difference of monocyte percentage in different disease activity groups.

### 3.4 ROC curve analysis of laboratory indicators and combined indicator for disease activity prediction in Crohn’s disease

The above indexes were included in the collinearity test. Results showed that the tolerance of all indexes were greater than 0. 10 and the variance inflation factors were less than 10, which represented that there was no multicollinearity in all indicators (S3 File).

Then, indexes were selected into the multivariate logistic regression model. Results showed that none of the above indexes could independently predict disease activity (p>0.05) (S4 File). The logistic regression model equation was established according to indexes and corresponding β coefficient. The equation is as follows: Logit (combined indicator) = 3.560 + (-0.001) * PLT + 0.118* Absolute neutrophil count +0.056 * PT + 0.016* CRP + (-0.093) * D-Dimer + 0.591* Absolute monocyte count + 0.031* ESR + (-0.065) * Hb + (-0.074) * ALB + 0.052* TBIL + 0.165* HCT. The p value of Hosmer-Lemeshow test is 0.184, which is greater than 0.05, indicating that the model fits well.

**Fig 3:**
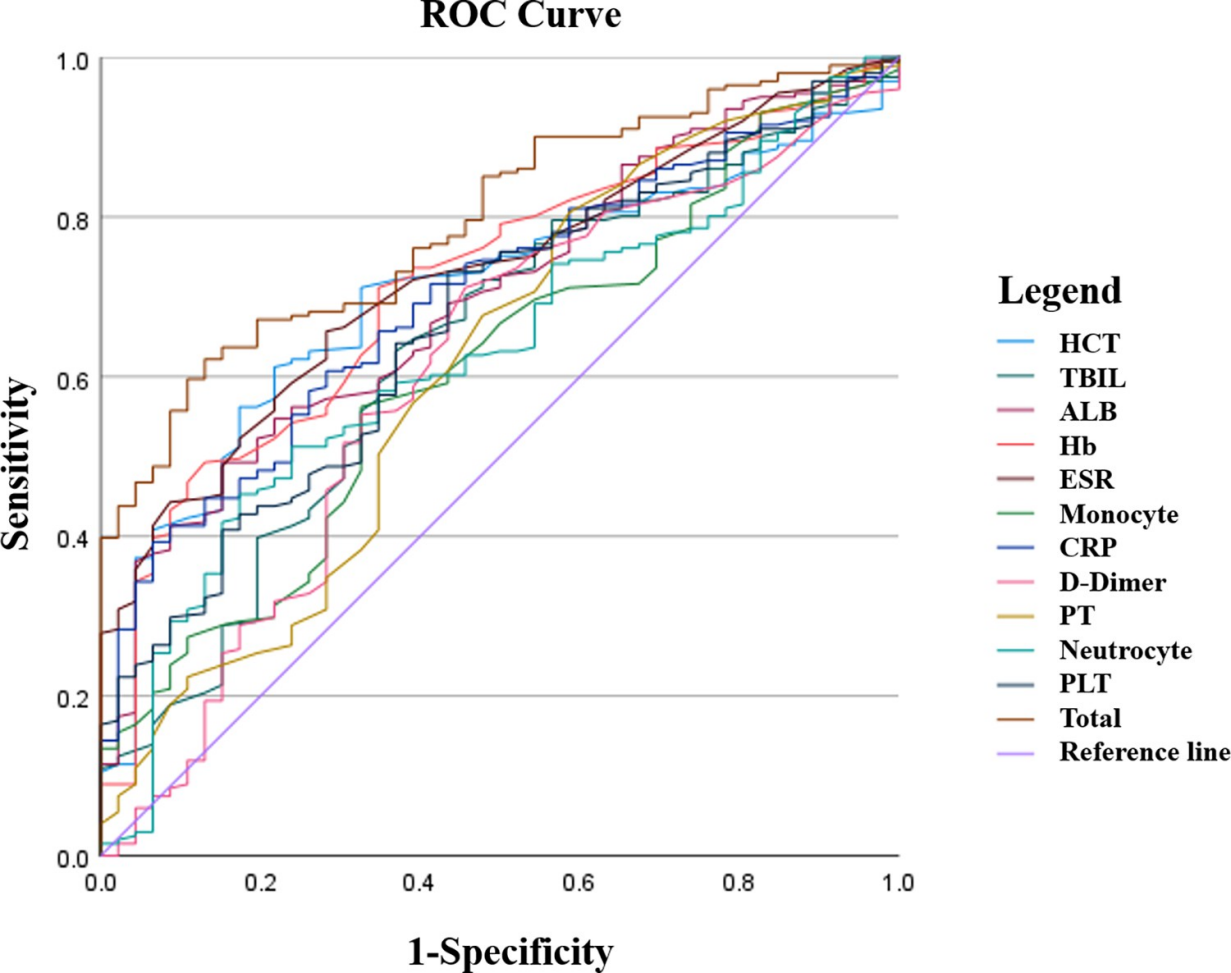
The ROC curves of the single indicator and the combined indicator to predict disease activity. Abbreviation: CDAI, Crohn’s Disease Activity Index; Hb, Hemoglobin; PLT, platelet count; HCT: Hematocrit; PT, prothrombin time; APTT, activated partial thromboplastin time; ESR, erythrocyte sedimentation rate; CRP, C-reactive protein, ALB, albumin; TBIL, total bilirubin.

The combined indicator was calculated according to the logistic regression model equation, and the ROC curves were drawn (Fig 3). Compared with the single indicator, the distinguished ability of the combined indicator to disease activity was significantly increased (p<0.05) (S5 File). It is concluded that a single laboratory indicator cannot independently predict disease activity, and the combined indicator established in this study has a stronger predictive ability of disease activity than a single indicator.

### 3.5 Value of absolute monocyte count and its percentage in predicting primary nonresponse in CD patients treated with anti-TNF-α drugs

To identify the predictive value of monocytes in combination with clinical and laboratory factors response to anti-TNF-α agents. We first explored the predictors of PNR to anti-TNF-α agents. Following univariate regression analysis based on 115 patients, we found that sex, BMI, Hb, HCT, CRP, ALB, APTT, and PT were correlated with PNR (p<0.2), while absolute monocyte count and monocyte percentage were not associated with PNR (p = 0.740 and p = 0.641) (S6 File).

### 3.6 The value of absolute monocyte counts and its percentage in predicting secondary non-response within 1 year in CD patients treated with anti-TNF-α drugs

Then, 78 patients with 1-year follow-up data were included in the analysis of SLOR of anti-TNF-α in CD patients. As expected, male, penetrating lesions, decreased hemoglobin content, decreased erythrocyte specific volume, increased platelet count, increased absolute monocyte count, increased monocyte percentage, increased CRP, increased ESR, decreased ALB, and decreased TBIL predicted SLOR within 1 year (Table 2). We included absolute monocyte count and monocyte percentage, respectively, in the multivariate analysis. The results showed that gender (OR = 0.06, p = 0.011), hemoglobin content (OR = 0.79, p = 0.04), and absolute monocyte count (OR = 411.9, p = 0.013) were independent predictors of SLOR within 1 year of receiving anti-TNF-α drugs in patients with CD. When multivariate logistic regression analysis included monocyte percentage and other laboratory indicators, only sex was an independent predictor of SLOR within 1 year in CD patients treated with anti-TNF-α drugs. Other laboratory indicators, including monocyte percentage, showed no predictive value for loss of response to anti-TNF-α drug therapy (Table 2).

**Table 2:** Monocyte percentage predict secondary loss of response at 1 year to anti-TNF-α in CD patients.

Characteristics	Univariate analysis		Multivariate analysis^#^		Multivariate analysis^&^	
	OR (95% CI)	p-value	OR (95% CI)	p-value	OR (95% CI)	p-value
Gender	0.28 (0.08–0.96)	0.043***	0.06 (0.01–0.51)	0.011***	0.13 (0.02–0.85)	0.033***
Age at disease onset	0.97 (0.91–1.03)	0.328				
Body mass index	1.01 (0.87–1.18)	0.857				
Location	1.04 (0.72–1.51)	0.822				
Stenosis	0.88 (0.27–2.92)	0.839				
Penetration	0.18 (0.02–1.43)	0.104*	0.25 (0.01–5.45)	0.377	0.19 (0.01–3.45)	0.259
Perianal lesions	0.63 (0.16–2.53)	0.513				
Absolute monocyte counts	7.74 (0.58–103.5)	0.122*	411.9 (3.50–48426.7)	0.013***		
Monocyte percentage	1.19 (0.96–1.47)	0.116*			1.45 (0.99–2.11)	0.054
CRP	1.02 (1.00–1.03)	0.081**	1.01 (0.98–1.04)	0.681	1.00 (0.97–1.03)	0.885
ESR	1.03 (1.01–1.06)	0.009***	1.02 (0.97–1.08)	0.359	1.01 (0.97–1.06)	0.574
ALB	0.88 (0.78–0.99)	0.030***	1.02 (0.87–1.21)	0.786	0.95 (0.80–1.14)	0.586
TBIL	0.81 (0.65–1.01)	0.062**	0.95 (0.72–1.26)	0.733	0.91 (0.67–1.23)	0.528
Hb	0.97 (0.94–0.99)	0.011***	0.79 (0.64–0.99)	0.040***	0.93 (0.76–1.14)	0.499
HCT	0.88 (0.80–0.98)	0.015***	1.93 (0.85–4.40)	0.118	1.13 (0.51–2.50)	0.763
PLT	1.00 (1.00–1.01)	0.100*	1.00 (0.99–1.00)	0.299	1.00 (0.99–1.01)	0.753
Neutrophils	1.13 (0.89–1.44)	0.319				
APTT	1.06 (0.94–1.19)	0.363				
PT	1.38 (0.82–2.35)	0.227				
D-Dimer	1.53 (0.60–3.91)	0.373				
Lymphocyte	2.02 (0.68–6.03)	0.208				
CDAI	1.00 (0.24–4.11)	1.000				

Note:

*** means p<0.05

** means p<0.1

* means p<0.2

#, multivariate regression analysis including absolute monocyte count; &, multivariate logistic regression analysis including monocyte percentage.

### 3.7 Nomogram establishment

**Fig 4:**
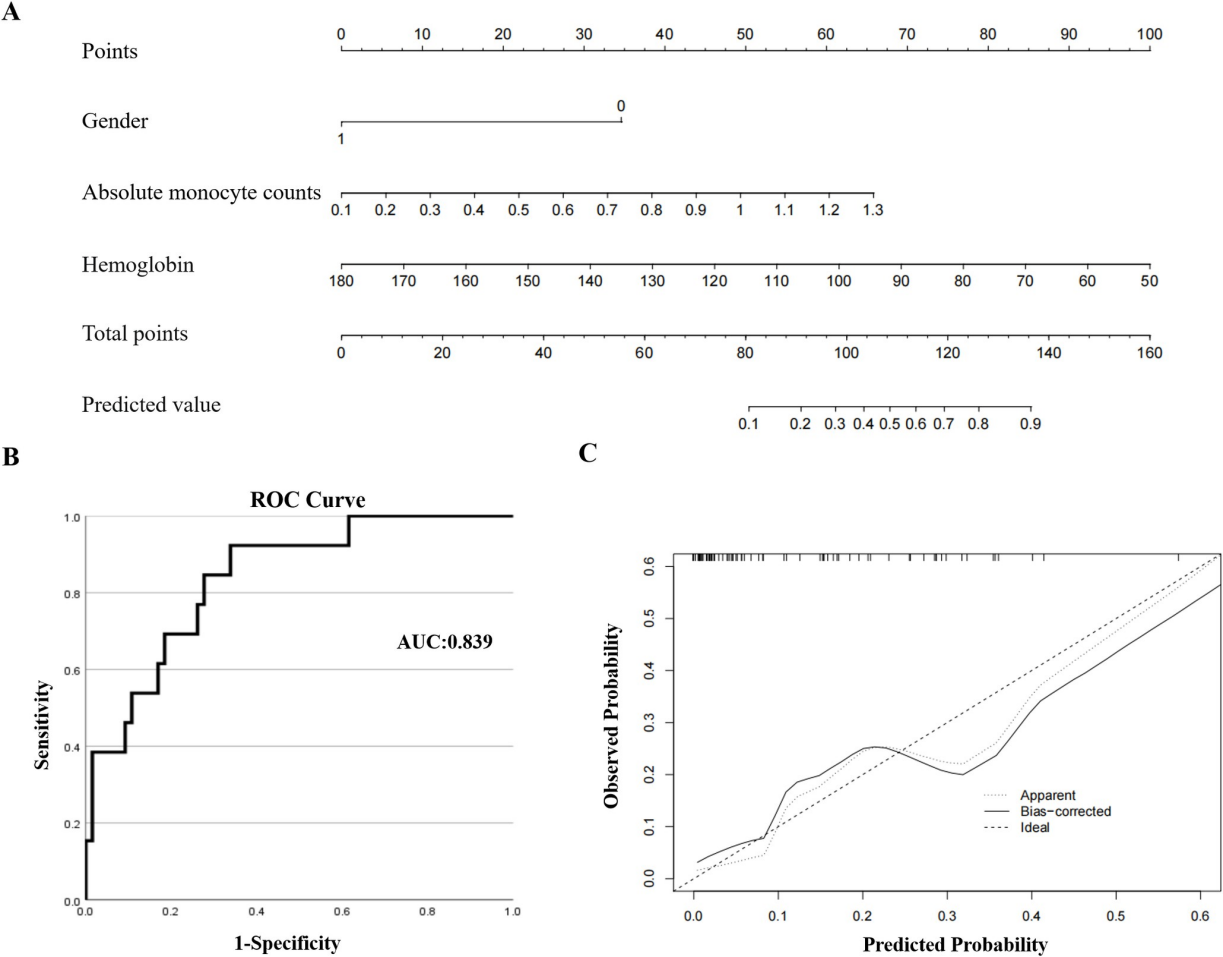
The nomogram for predicting secondary loss of response within 1 year in Crohn’s Disease patients treated with anti-TNF-α drugs. (A) A nomogram based on gender, absolute monocyte counts, and hemoglobin; (B) The ROC curve of predicted value based on nomogram; (C) Calibration curve of the nomogram.

In order to accurately predict the SLOR within 1 year of anti-TNF-α agents in CD patients, we constructed a nomogram based on gender, absolute monocyte counts, and hemoglobin (Fig 4A). The area under the ROC curve of predicted value was 0.893, which meant that the model has a good differentiation ability (Fig 4B). Fig 4C showed the calibration curve of model, which presented the good calibration of nomogram. P value of Hosmer-Lemeshow was 0.919, greater than 0.05, indicating there was no statistically significance between observed value and predicted value.

## 4. Discussion

This is the first study to evaluate monocyte related indicators on the identification of disease activity and response to biologics treatment in patients with CD. Results demonstrated that absolute monocyte count was strongly associated with disease activity in patients with CD and predicted SLOR within 1 year of anti-TNF-α treatment, but not PNR. In contrast, no relationship was found between monocyte percentage and disease activity or treatment effect of biologics. Finally, a nomogram based on gender, absolute monocyte counts, and hemoglobin showed a good differentiation and calibration ability to predict the SLOR within 1 year of anti-TNF-α agents in CD patients.

At the time of diagnosis and treatment of CD, disease activity needs to be assessed. Colonoscopy is considered the gold standard for Crohn’s disease activity evaluation [27]. However, due to the poor economic conditions and poor compliance of some patients, colonoscopy is not easily available. Therefore, it is feasible to find easy-to-obtain and affordable indicator derived from blood samples, which can evaluate disease activity easier and faster. Previous studies have indicated that various indicators were related to disease activity, such as ESR, CRP, neutrophils, and FC [28,29]. In our study, we found that absolute monocyte counts related to disease activity, while monocyte percentage was not. Previous studies have shown that proinflammatory CD16+ monocytes in peripheral blood are significantly increased in active CD. In the lamina propria of intestinal mucosa in patients with Crohn’s disease, CD16+ monocytes are important proinflammatory cells [30]. Therefore, monocytes are closely related to disease activity. The reason why monocyte percentage was not related to disease activity is that monocyte percentage is the proportion of monocytes to white blood cells, so it is affected by neutrophils, lymphocytes, etc. In the course of IBD, neutrophils are uncontrolled recruited to colon [31]. Neutrophil activation in mucous membranes reflects disease activity [32]. Multiple studies have shown that neutrophil-lymphocyte ratio is a marker of disease activity and severity [33,34]. Therefore, absolute monocyte counts more clearly reflects disease activity. In addition, our study showed that a single laboratory indicator cannot independently predict disease activity, and the combined indicator based on multiple indicators related to disease activity in this study has a significantly higher ability to distinguish disease activity compared with a single indicator.

Anti-TNF-α agents are most widely used in patients with CD [35], but some patients do not respond to IFX in the initial or later stages of treatment. However, the underlying mechanism remains unknown. Potential predictors of response to infliximab have been evaluated and summarized as patient-related factors, disease-related factors and treatment-related factors [36]. In CD, activated monocytes drive the inflammatory process by releasing pro-inflammatory cytokines and chemokines. As monocytes could produce TNF-α, it plays a central role in intestinal inflammation in patients with CD and is predictive of anti-TNF-α therapeutic response [19,37]. Studies showed that compared with responders, cytokines such as TNF-α, IL-23, and IL-8 are up-regulated in mononuclear cells of non-responders treated with IFX, indicating cytokine profiles of monocytes are predictive markers of IFX response [35]. Another study showed that TNF-α greater than 500 pg/ml was an independent predictor of 6-week clinical response to infliximab treatment [38]. Therefore, in our study, we studied the predictive value of monocytes to anti-TNF-α therapy. Our study pointed out that absolute monocyte counts and monocyte percentage could predict SLOR within 1 year of treatment, but not PNR in univariate regression analysis. Further multivariate regression analysis indicated that only absolute monocyte counts was an independent predictor of SLOR at 1 year, and that monocyte percentage had no predictive value for loss of response to anti-TNF-α therapy. The monocyte percentage is affected by the number of neutrophils, lymphocytes, eosinophils and other white blood cells, so its percentage cannot reflect the number of monocytes well, nor can it accurately reflect the disease activity and treatment effect. It has been reported that patients with active CD treated with infliximab induced monocyte apoptosis, which exerted an anti-inflammatory effect [39]. This is consistent with our results that when the therapeutic effect of anti-TNF-α drugs on CD patients is decreased, the effect of inducing monocyte apoptosis is weakened, and the proportion of monocytes is increased. Therefore, the increase of absolute monocyte counts reflects the loss of secondary response to anti-TNF-α drugs to some extent. Marileen M Prins et al. have shown that the expression level of TREM-1 in CD14+ monocytes is one of the factors affecting response of anti-TNF-α therapy [40]. The reason of predictive value difference of monocyte between PNR and SLOR is not clear. We speculate that changes of monocytes activity and TNF-α cytokines level in the course of disease lead to this difference. Further studies are expected to elucidate the mechanism.

Several limitations must be addressed. Firstly, our study was retrospective and some data about treatment response were lost. Secondly, even though we studied the predictive value of monocytes in the therapeutic effect of infliximab and Adalimumab, while not explored in vedolizumab and ustekinumab because of the limited samples. Thirdly, this study only aimed at monocyte counts before treatment. Analysis of monocyte counts collected at multiple time points may provide the best predictor.

In conclusion, absolute monocyte counts could be good biomarkers for disease activity of patients with CD. Most importantly, serves as a promising biomarker in the prediction of response to anti-TNF-α therapy in CD patients with maintenance treatment. It helps to guide the most appropriate treatment for CD patients and reducing medical costs.

## Supporting information

S1 File Correlation analysis of monocyte percentage and disease activity. (DOCX)

S2 File Relationship between absolute monocyte counts or monocyte percentage and different activity groups. (TIF)

S3 File Results of collinearity test. (DOCX)

S4 File Multivariate logistic regression analysis to explore indicators predicting disease activity. (DOCX)

S5 File Area under curve of ROC curves. (DOCX)

S6 File Predictors of primary nonresponse to anti-TNF-α in CD patients. (DOCX)
