# Supplementary material for: Absolute monocyte counts could predict disease activity and secondary loss of response of patients with Crohn’s disease treated with anti-TNF-α drug
Source: PLoS One. 2024 Apr 10;19(4):e0301797. doi: 10.1371/journal.pone.0301797 (PMC11006187; doi:10.1371/journal.pone.0301797)
Supplement: S1 File — (DOCX) [file pone.0301797.s001.docx]

| Supplementary material 1. Correlation analysis of monocyte percentage and disease activity | | |
| --- | --- | --- |
|  | R | p |
| CRP | 0.174 | 0.003 |
| ESR | 0.038 | 0.529 |
| ALB | -0.161 | 0.007 |
| TBIL | -0.057 | 0.339 |

Abbreviation: CRP, C-reactive protein; ESR, erythrocyte sedimentation rate; ALB, albumin; TBIL, total bilirubin.
