# Supplementary material for: Absolute monocyte counts could predict disease activity and secondary loss of response of patients with Crohn’s disease treated with anti-TNF-α drug
Source: PLoS One. 2024 Apr 10;19(4):e0301797. doi: 10.1371/journal.pone.0301797 (PMC11006187; doi:10.1371/journal.pone.0301797)
Supplement: S3 File — (DOCX) [file pone.0301797.s003.docx]

| Supplementary material 3. Results of collinearity test | | |
| --- | --- | --- |
| Indicators | Collinearity test | |
|  | Tolerance | Variance Inflation Factor |
| Hemoglobin | 0.168 | 5.970 |
| Platelet count | 0.547 | 1.828 |
| Absolute neutrophil count | 0.572 | 1.747 |
| Prothrombin time | 0.876 | 1.142 |
| C-reactive protein | 0.423 | 2.365 |
| Albumin | 0.629 | 1.589 |
| Total bilirubin | 0.804 | 1.243 |
| Absolute monocyte count | 0.476 | 2.100 |
| Hematocrit | 0.189 | 5.297 |
| Erythrocyte sedimentation rate | 0.428 | 2.337 |
| D-Dimer | 0.955 | 1.047 |
