# Supplementary material for: Absolute monocyte counts could predict disease activity and secondary loss of response of patients with Crohn’s disease treated with anti-TNF-α drug
Source: PLoS One. 2024 Apr 10;19(4):e0301797. doi: 10.1371/journal.pone.0301797 (PMC11006187; doi:10.1371/journal.pone.0301797)
Supplement: S4 File — (DOCX) [file pone.0301797.s004.docx]

| Supplementary material 4. Multivariate logistic regression analysis to explore indicators predicting disease activity. | | |
| --- | --- | --- |
| Indicators | Multivariate logistic regression | |
|  | β | p |
| Platelet count | -0.001 | 0.644 |
| Absolute neutrophil count | 0.118 | 0.408 |
| Prothrombin time | 0.056 | 0.753 |
| C-reactive protein | 0.016 | 0.311 |
| D-Dimer | -0.093 | 0.194 |
| Absolute monocyte count | 0.591 | 0.704 |
| Erythrocyte sedimentation rate | 0.031 | 0.086 |
| Hemoglobin | -0.065 | 0.072 |
| Albumin | -0.074 | 0.057 |
| Total bilirubin | 0.052 | 0.191 |
| Hematocrit | 0.165 | 0.202 |
| Constant | 3.560 | 0.245 |
