# Supplementary material for: Absolute monocyte counts could predict disease activity and secondary loss of response of patients with Crohn’s disease treated with anti-TNF-α drug
Source: PLoS One. 2024 Apr 10;19(4):e0301797. doi: 10.1371/journal.pone.0301797 (PMC11006187; doi:10.1371/journal.pone.0301797)
Supplement: S5 File — (DOCX) [file pone.0301797.s005.docx]

| Supplementary material 5. Area under curve of ROC curves | | |
| --- | --- | --- |
| Indicaters | AUC | p |
| D-Dimer | 0.598 | <0.05 |
| Absolute monocyte count | 0.607 | <0.05 |
| Prothrombin time | 0.608 | <0.05 |
| Absolute neutrophil count, | 0.623 | <0.05 |
| Total bilirubin | 0.633 | <0.05 |
| Platelet count | 0.677 | <0.05 |
| Albumin | 0.695 | <0.05 |
| C-reactive protein | 0.699 | <0.05 |
| Hematocrit | 0.717 | <0.05 |
| Hemoglobin | 0.729 | <0.05 |
| Erythrocyte sedimentation rate | 0.731 | <0.05 |
| Composite Indicators | 0.813 | Reference |
