# Supplementary material for: Absolute monocyte counts could predict disease activity and secondary loss of response of patients with Crohn’s disease treated with anti-TNF-α drug
Source: PLoS One. 2024 Apr 10;19(4):e0301797. doi: 10.1371/journal.pone.0301797 (PMC11006187; doi:10.1371/journal.pone.0301797)
Supplement: S6 File — (DOCX) [file pone.0301797.s006.docx]

| Supplementary material 6. Predictors of primary nonresponse to anti-TNF-α in CD Patients | | |
| --- | --- | --- |
| Characteristics | Univariate analysis | |
|  | OR (95% CI) | p-value |
| Gender | 0.34 (0.10-1.19) | 0.092** |
| Age at disease onset | 0.98 (0.91-1.04) | 0.437 |
| Body mass index | 0.86 (0.70-1.05) | 0.135* |
| Location | 1.19 (0.83-1.71) | 0.335 |
| Stenosis | 0.50 (0.14-1.83) | 0.299 |
| Penetration | 1.73 (0.47-6.35) | 0.406 |
| Perianal lesions | 0.58 (0.15-2.30) | 0.437 |
| Absolute monocyte counts | 0.57 (0.02-15.95) | 0.740 |
| Monocyte percentage | 0.93 (0.70-1.24) | 0.641 |
| CRP | 1.02 (1.00-1.04) | 0.013*** |
| ESR | 1.02 (0.99-1.04) | 0.238 |
| ALB | 0.88 (0.79-0.97) | 0.011*** |
| TBIL | 0.97 (0.85-1.11) | 0.647 |
| Hb | 0.98 (0.95-1.00) | 0.074** |
| PLT | 1.00 (1.00-1.01) | 0.249 |
| Absolute neutrophils counts | 1.00 (0.75-1.33) | 0.991 |
| APTT | 1.07 (0.97-1.19) | 0.180* |
| PT | 1.68 (1.03-2.72) | 0.037*** |
| D-Dimer | 0.30 (0.02-4.40) | 0.379 |
| Absolute lymphocyte counts | 0.72 (0.19-2.66) | 0.619 |
| HCT | 0.92 (0.84-1.01) | 0.095** |
| CDAI | 3.15 (0.39-25.67) | 0.285 |

Note: ***represents p value <0.05; ** represent p value <0.1; * represent p value <0.2.

Abbreviation: CD, Crohn's disease; Anti-TNF-α, Anti-tumor necrosis factor α; HR: Hazard ratio; CI, Confidence interval; Hb, Hemoglobin; PLT, Platelet count; HCT: Hematocrit; PT, Prothrombin time; APTT, Activated partial thromboplastin time; ESR, Erythrocyte sedimentation rate; CRP, C-reactive protein, ALB, Albumin; TBIL, Total bilirubin; CDAI, Crohn’s Disease Activity Index.
